# The Difference LTC Corporate Ownership Makes in Collaborations With Emergency Operation Centers During Disasters

**DOI:** 10.1093/geroni/igab046.974

**Published:** 2021-12-17

**Authors:** Debra Dobbs, Joseph June, David Dosa, Lindsay Peterson, Kathryn Hyer

**Affiliations:** 1 University of South Florida, School of Aging Studies, University of South Florida, Florida, United States; 2 University of South Florida, Tampa, Florida, United States; 3 Brown University, Providence, Rhode Island, United States; 4 University of South Florida, Bradenton, Florida, United States; 5 University of South Florida, University of South Florida, Florida, United States

## Abstract

Collaboration between nursing homes (NHs) and assisted living communities (ALCs) with state and local entities (e.g., emergency operation centers (EOCs)) is critical during a disaster. The corporate structure of NHs and ALCs can make a difference in their ability to collaborate with these entities during a disaster. This mixed-method study examines differences in satisfaction with collaboration with state and local entities during Hurricane Irma in Florida in 2017 between corporate-owned NHs (N=24), larger (25+ beds) ALCs (N=38) and smaller ALCs (N=30). We also explore collaboration in Florida NHs (N=35) and ALCs (N=123) specific to COVID19. Scaled 1-5 survey data results indicate that small ALCs are the least satisfied (M=2.90) with EOC collaboration, compared to NHs (M=3.04) and larger ALCs (M=3.33) during Irma. Smaller ALCs were more dissatisfied with COVID19 mandates compared to larger ALCs and NHs. Ways to improve collaboration during a disaster, especially for smaller ALCs, will be discussed.

